# Role Stress and Turnover Intention of Front-Line Hotel Employees: The Roles of Burnout and Service Climate

**DOI:** 10.3389/fpsyg.2020.00036

**Published:** 2020-01-30

**Authors:** Biyan Wen, Xiaoman Zhou, Yaou Hu, Xiao Zhang

**Affiliations:** School of Management, Jinan University, Guangzhou, China

**Keywords:** role stress, burnout, service climate, turnover intention, front-line hotel employees

## Abstract

High turnover rate represents one of the most significant challenges the hotel industry faces. High turnover rates mean labor shortages, resulting in high costs of recruiting, staffing and training. Turnover also has a negative impact on service quality. Scholars continue to search for the root causes of turnover and propose solutions. To further understand employees’ turnover intention, this study reveals the role of stress on hotel front-line employees’ turnover intention through the mediation of burnout. Moreover, the study examines the moderating effect of service climate on the underlying mechanism that links role stress with turnover intention. Using a sample of 583 questionnaires from front-line hotel employees in South China, this study reveals that role stress as a four-dimensional construct (i.e., conflict, ambiguity, qualitative overload and quantitative overload) has a statistically significant impact on burnout, which leads to turnover intention. Burnout completely mediates the relationship between role stress and turnover intention, that is, employees under role stress do not resign immediately unless they experience high levels of burnout. In addition, service climate moderates the influence of role stress on burnout, suggesting a moderated mediation relationship. The study contributes to the organizational management literature by confirming the four dimensions of role stress and demonstrating how role stress impacts employees’ turnover intention. Furthermore, the critical effect of service climate is further investigated. Theoretical contributions and managerial implications are discussed based on the findings. the study also investigates the moderating effect of service climate on role stress (challenge-hindrance stressors) and burnout.

## Introduction

The hotel industry is facing a labor shortage in developed countries as well as emerging markets (Kim W.G. et al., 2016). The total turnover rate in the hospitality industry was 27.6 percent in 2014 in China alone (Chen et al., 2016), generating attention from academics and industry managers (Mohsin et al., 2013). Therefore, reducing turnover rate is a priority for the industry.

Front-line hotel employees play an essential role in creating positive customer experiences, which are key factors of customer satisfaction and appraisal of service quality (Wirtz and Jerger, 2016; Ayşe Banu and Alexander Ellinger, 2018). Hotel employees also face many challenges while performing their jobs (Kim et al., 2015). Facing a strenuous workload, frequent changes of circumstances, lack of performance feedback and low pay, they are easily irritated and exhausted, which in turn affects their behavior and may lead to resignation (Kim et al., 2012).

One critical challenge faced by front-line employees in the hotel industry is role stress. Long hours of operation, polychronic behaviors, a high demands-low resources job model and little feedback result in high levels of stress and burnout (O’ Neill and Davis, 2011). Over time, role stress and burnout cause poor job performance and high turnover intention. Employee role stress has intrigued scholars for the past two decades, but studies mainly focus on such fields as nursing, accounting and sales (Jaramillo et al., 2006; Kelly and Barrett, 2011; Kuo et al., 2014). The hotel industry, although it is perceived as more stressful, has drawn less attention (Jones et al., 2007; Cho et al., 2009). Therefore, to address this limitation in literature, this study examines role stress in the context of hotel industry.

Role stress can exert complicated effects in organizations. A reasonable and tolerable level of stress can generate positive effects, such as higher motivation, a competitive edge and a dynamic working atmosphere (Beehr and Newman, 1978). Conversely, excessive stress that causes burnout has negative impacts for both organizations and individuals. This argument is in line with the challenge-hindrance theory of work stressor (Cavanaugh et al., 2000), which suggests that challenge stressors may produce positive emotions, even though employees may feel stressed. On the contrary, hindrance stressors are often associated with egregious or undesirable constraints, job demands and work circumstances, causing negative outcomes. Challenge stressors may produce positive emotions, even though employees may feel stressed (Montani et al., 2017; Karatepe et al., 2018).

With researchers recognizing the importance of role stress in organizational management, the components of role stress are still under debate. Most studies focus on role conflict and role ambiguity, and neglect role overload, a separate but correlated dimension of role stress (Kahn et al., 1964; Wirtz and Jerger, 2016). Furthermore, as an important dimension of role stress, the conceptualization of role overload is inconsistent. According to some scholars (e.g., Cooper and Marshall, 1976; Jung et al., 2012), role overload is regard as a quantitative factor, while role conflict and ambiguity are qualitative factors. Others believe that role overload consists of both quantitative and qualitative overload (e.g., Kelly and Barrett, 2011). One criticism of much empirical research on role stress is that scholars have paid little attention to examining the effects of quantitative and qualitative role overload. In addition, the concepts of role conflict and role overload have been often used interchangeably, although they actually represent two distinct dimensions (Hecht, 2001). Because role conflict and role overload are derived from different contextual sources and contribute to different physical and psychological consequences, the dimensions of role overload need to be further explored.

When stress exceeds a threshold, psychological or behavioral reactions will be triggered for defense (Jaramillo et al., 2011). According to person-environment fit theory, employee’s evaluation of the organizational climate is important in the process of managing stress, which is considered one of the principal mechanisms linking stress to related consequences (Webster et al., 2011). This study argues that service climate moderates the effect of role stress on turnover intention through the mediation of burnout. Specifically, if hotels provide a strong support system to help workers relieve pressure, dispel negative emotions from work and adjust to the organization, employees are likely to show better performance and higher loyalty, thus reducing the turnover rate.

The study aims to enrich role stress and service management literature in four ways. First, previous research on role stress has focused on role conflict and ambiguity, but rarely discussed the effects of quantitative and qualitative role overload. This study contributes to the stress literature by demonstrating the four dimensions of role stress. Second, this study explores the underlying mechanism that links role stress with turnover intention, and clarifies the mediating effect of burnout. Third, the study demonstrates how employee perceptions of service climate moderates the impact of role stress (challenge-hindrance stressors) on burnout. Finally, addressing the limitation in past research that no consensus is reached in the attitudinal and behavioral outcomes of challenge-hindrance stressors, this study makes an exploratory research about challenge-hindrance stressors by identifying the impacts of these two stressors on burnout and turnover intention.

## Literature Review and Hypotheses

### Role Stress

Kahn et al. (1964) introduced the concept and development process of role stress, arguing that role stress is the result of communication and interaction between role senders and receivers. This process is influenced by such factors as enterprise systems, external business environment and personal factors. According to classical organization theory, every position in a formal organizational structure should have specified tasks or responsibilities. However, in reality, employees often have a variety of roles and expectations, requirements and standards of evaluation from others.

Role theory states that when the expectations and requests of an employee are inconsistent, he/she may feel stressful, become dissatisfied, and perform less efficiently. Therefore, role conflict can be seen as the result of conflicting expectations imposed on individuals (Rizzo et al., 1970). Role theory states that role ambiguity refers to a lack of necessary information required for a given position, which may lead to a person being discontented with his role, experiencing anxiety and misrepresenting reality. Rizzo et al. (1970) developed and tested role conflict and ambiguity, and showed that these two constructs are factorially identifiable and independent. Role ambiguity and role conflict are two important factors influencing employee satisfaction and turnover intention in the hospitality industry (Kemery et al., 1985; Kim and Lee, 2009).

Scholars have not reached a consensus on the dimensions of role stress. Some propose that role stress has a two-dimensional structure consisting of conflict and ambiguity (Walker et al., 1975), while others see role stress as a three-dimensional structure consists of role conflict, ambiguity and overload (Abdel-Halim, 1981; Schaubroeck et al., 1989). Some scholars believe that role overload is a part of role conflict (Coverman, 1989), while others regard role overload as a separate dimension (Peterson et al., 1995). According to Jones et al. (2007), role overload is a sensation that job requirements are overpowering relative to usable capabilities and resources. Moreover, the effect of role overload appears to be subsumed by role stressors and is conceptually different from two other stressors: role conflict and ambiguity (Singh, 1993). Role overload effects employee well-being negatively. As employees become overloaded, their physical and psychological health may decline. Other unfavorable outcomes of role overload contain low organizational commitment, high absenteeism and turnover intention (Jones et al., 2007; Mulki et al., 2008; Huang et al., 2018).

There are also arguments regarding the dimensionality of role overload. Some researchers believe that role overload comprises two independent factors. They consider lack of time as quantitative role overload and employees exceeding high requirements as qualitative role overload. However, empirical research on role stress has not tested quantitative and qualitative role overload sufficiently (Kelly and Barrett, 2011; Jung et al., 2012).

The subjects of our study are front-line employees in the hotel industry, where extensive workloads are a common feature of their job. During very busy hours, they sometimes cannot complete every task well because of too much work or time limits, and then role overload may occur. Thus, role overload is an independent assessment considering the specific work of front-line employees in the hotel industry. Therefore, this study adopts the view that role stress has four aspects: conflict, ambiguity, quantitative overload and qualitative overload.

Interest in the complexities of the challenge-hindrance stressors factors has increased in recent years. Some scholars suggest that work overload and responsibility are challenge stressors, and that role ambiguity and conflict are hindrances (Lepine et al., 2005; Crawford et al., 2010; Karatepe et al., 2014). Previous literature suggests that challenge stressors have favorable effects on job outcomes, such as work engagement and job satisfaction, while hindrances may lead to negative outcomes (Lepine et al., 2005; Karatepe et al., 2014). However, some researchers argue that challenge stressors (i.e., work overload) can also lead to detrimental results (Ahuja et al., 2007; Karatepe, 2013). Although different types of stressors have been identified, theoretical debate and inconsistency of findings regarding the attitudinal and behavioral outcomes of challenge and hindrance stressors merit further study (Webster et al., 2011; Karatepe et al., 2014).

### Burnout

Freudenberger (1974) who proposed the burnout concept, believes it has two aspects: physical and behavioral symptoms. Physical symptoms refer to the individual’s perceived state of fatigue and depletion, and behavioral symptoms mean that it is difficult to control emotions, eventually leading to poor performance. Schaufeli et al. (1996) and Maslach et al. (2001) conducted a series of studies on the connotation and measurement of burnout in the service industry. Specifically, burnout is defined as the serious psychological and physical symptom resulting from continuous stressful and frustration at work (Maslach and Jackson, 1984).

The components of burnout are controversial (Maslach and Jackson, 1984; Demerouti et al., 2001; Densten, 2001), although most studies agree that burnout results from three factors: emotional exhaustion, depersonalization and lack of self-efficacy. Measurement of burnout has also been debated, with many scholars advocating a two-dimensional framework based on emotional exhaustion and depersonalization. Case and empirical studies seem to confirm that emotional exhaustion and depersonalization are the core factors of burnout (e.g., Demerouti et al., 2001; Kilroy et al., 2016).

Some researchers explore why self-efficacy is rather ambiguous. First, self-efficacy is an aspect of personal character rather than a response to tension (Demerouti et al., 2001; Kim S.M. et al., 2016). Second, self-efficacy is believed to be independent from emotional exhaustion and depersonalization, and a meta-analysis supported this view (Lee and Ashforth, 1996; Büssing and Glaser, 2000). Third, the statement-of-self-efficacy-scale is positive while emotional exhaustion and depersonalization are negative (For the self-efficacy subscale only, high scores represent low burnout), which may cause inconsistencies in measurement.

With stressful working situations caused by high customer-to-staff interactions, polychronic behaviors, and long working hours, burnout is prevalent in the hotel industry. In consideration of the specific research context of this study, the authors concentrate on emotional exhaustion and depersonalization these two core factors of burnout.

### Role Stress and Burnout

Some empirical research shows a positive relationship between role stress and burnout (Bacharach et al., 1991; Iverson et al., 1998; Thomas and Lankau, 2009). For instance, Maslach and Jackson (1984) reported that burnout was caused by role overload. In terms of the effects of specific dimensions of role stress on burnout, role conflict and role ambiguity are found to be significantly related to burnout (Brookings et al., 1985). Role conflict and role overload are found to influence burnout and job satisfaction directly, while role overload is found to be a precursor to depersonalization (Bacharach et al., 1991).

Role conflict and ambiguity have positive influences on emotional exhaustion and non-individuation, while ambiguity has a negative impact on personal accomplishment (Karatepe and Uludag, 2008). Konstantinou et al. (2018) find that role conflict is an important predictor of emotional exhaustion and depersonalization, and role ambiguity positively relates with lack of self-efficacy. Additionally, job stressors are found to be positively related to emotional exhaustion among hotel employees (Darvishmotevali et al., 2017). Employees who have work overload and work-family conflicts are more likely to experience emotionally exhausted; and high levels of emotional exhaustion may result in employee withdrawal intentions (Karatepe, 2013). Previous research provided evidence on the effect of role stress on burnout and turnover intention. However, this stream of research examined only some dimensions of role stress and did not make a further distinction about role overload (e.g., Jung et al., 2012).

Burnout occurs more commonly among hotel employees who serve many customers but have difficulties coping with excessive emotional and communication demands as well as long-term frustration because of limited emotional resources (Teoh et al., 2019). Choi et al. (2019) confirm that job stress is a significant antecedent of hospitality employees’ burnout and turnover intention. Based on the literature, the authors posit the hypothesis:

**H1:** *Role stress has a significant impact on burnout.*

### Burnout and Turnover Intention

According to transactional theory of stress (Lazarus and Folkman, 1984) and empirical evidences, stressful tasks and working environment are potential causes of turnover (Jaramillo et al., 2006; Karatepe and Karatepe, 2010; Jung et al., 2012). When hotel employees experience excessive role stress and cannot obtain the resources to relieve it, they will consider coping the depressed situation with turnover.

When burnout accumulates and exceeds a tolerable range, employees may attribute such uncomfortable experiences to a mismatch with the organization (Bliese and Jex, 2002). Once employees feel that the situation cannot be improved, and they cannot access the resources to ameliorate the condition, their attitudes and behaviors may be affected (Kristof, 1996). In the long run, employees may consider leaving to achieve a better balance.

The positive effect of burnout on turnover intention has been proved certainly by many scholars (e.g., Kim and Stoner, 2008; Kim and Lee, 2009; Kim S.M. et al., 2016), and they highlight that burnout correlates with job dissatisfaction, absenteeism and turnover (Magnano et al., 2017; Mérida-López and Extremera, 2017). Burnout strongly affects work outcome variables, and the combined impact of burnout and role stress is more obvious while burnout plays a strong intermediary role between stress variables and work outcome variables (Singh, 1993). Previous research has established the effect of burnout on turnover intention (Kim and Stoner, 2008; Kim and Lee, 2009; Lingard, 2010) and the mediation effect of burnout on the relationship between work overload and work-family conflict on turnover intention (Ahuja et al., 2007). On the basis of these considerations, the authors posit the following two hypotheses:

**H2:** *Burnout has a significant positive impact on employee turnover intention.*
**H3:** *Burnout mediates the relationship between role stress and employee turnover intention.*

### Moderating Effects of Service Climate

The research about organizational service climate has dramatically confirmed the importance of creating a good organizational climate (Jung and Yoon, 2013; Chang, 2016; Lin and Liu, 2016). In recent years, interest in creating a positive service climate as a way to improve employee attitudes, behaviors and performance has increased. Service climate refers to “perceptions of the events, practices, procedures, and behaviors that are rewarded, supported and expected in a customer service setting” (Schneider et al., 1998). According to the definition, service climate is different from general climate because it is a strategical goal for improving service quality (Schneider et al., 1998; Hong et al., 2013). Hotels are supposed to provide necessary technical and information support for employees and establish smooth communication systems to create good service climates.

According to the person-environment fit theory (Kristof, 1996; Cable and Parsons, 2001), individual attitudes and behaviors are significantly influenced by how employees perceive the organizational climate. A positive service climate aligns employee attitudes and behaviors with organizational strategy and facilitates achieving service quality goals (Kralj and Solnet, 2010). Positive service climate lets employees feel a sense of attachment to the organization, improves their work engagement, and have a moderating effect on stress consequences, according to several researchers (Kim et al., 2012; Jung and Yoon, 2013; Arasli et al., 2017). When employees’ perceived organizational service climate is favorable, they can deal with the difficulties of the job and reduce role stress and burnout more effectively (Johnson, 1996).

In hotel industry, employees perceive service climate to be framed by organizational supports and culture for employees to provide quality services (Hong et al., 2013). When employees find that the hotels lack guidelines to support them cope with role stress and fail to communicate with them adequately, they are likely to become burnout. Conversely, positive service climate will help employees answer the questions “what should I do?” and “How to accomplish it?” (Jaramillo et al., 2006), thus lessening the negative impact of role stress on burnout. Accordingly, the authors propose:

**H4:** *Employees’ perceived organizational service climate moderates the relationship between role stress and burnout.*
**H5:** *Employees’ perceived organizational service climate moderates the indirect effect of role stress on turnover intention via burnout such that the indirect effect is stronger when service climate is low rather than high.*

**FIGURE 1:**
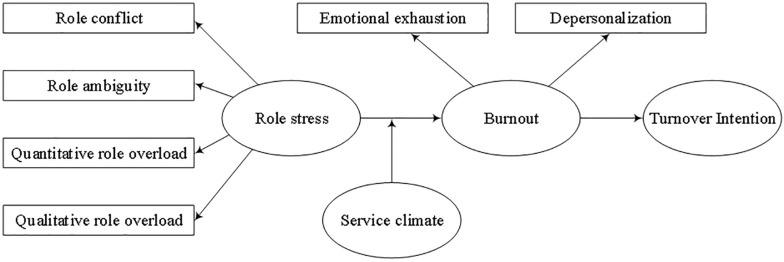
Research model.

Thus, the relations among these hypotheses arevisualized in Figure 1.

## Research Methodology

### Ethics Statement

The study belongs to National Natural Science Foundation of China (NSFC), which has been strictly assessed and proved by NSFC. An additional ethics approval was not required as per applicable institutional and national guidelines and regulations. However, this study was reviewed and approved by the School of Management of Jinan University. All participants (employees and their managers) provided written informed consent, and they were informed of their right to withdraw from the survey at any time. Also, we assured them that all information provided would be protected under strict confidentiality rules.

### Participants

The respondents came from different hotel departments, including the front office, food and beverage, housekeeping, bodybuilding and sports entertainment and marketing. In the pilot study, 53.6 percent of respondents were female and 46.4 percent male, while 64.1 were female and 35.9 percent male in the main study. Two age groups predominated: 18–22 years old (30.2%) and 36–45 years old (24%) in the pilot study, and 23–28 years old (54.2%) and 18–22 years old (24%) in the main study. Most respondents had lower education (i.e., college or vocational school). 69.3 percent of the respondents were full-time employees while 17.9 percent were interns. The monthly income of front-line employees is usually low, and 83.3 percent of them made less than 3000 RMB. In the pilot study, the tenure of respondents in the hotel industry was between 7 months to 5 years (for 44.7%). In the main study, about half (for 49.5%) of the employees working in hotels had tenures between 7 months to 2 years.

### Measures

#### Role Stress

Role stress was assessed with two dimensions proposed by Rizzo et al. (1970): role conflict (10 items) and role ambiguity (5 items). This scale shows high reliability and validity in many empirical studies. We also adopt the role overload scale (10 items) developed by Ivancevich and Matteson (1980). Sample items include “I receive an assignment without the manpower to complete it”; “I feel certain about how much authority I have”; “I am responsible for a number of projects or tasks”; and “My work seems to be getting more and more complicated.” All variables in the study were measured on 7-point Likert type scales (1 = “not at all,” and 7 = “extremely”).

#### Burnout

Since the subjects of this research are front-line employees in the hotel industry, the authors utilize the MBI-General Survey (Schaufeli et al., 1996) with 11 items to assess emotional exhaustion and depersonalization. Sample items include “I feel burned out from my work”; and “I have become more cynical about whether my work contributes anything.”

#### Organizational Service Climate

The authors used the global service climate scale which consists of seven items (Schneider et al., 1998) to measure organizational service climate. Sample items include “My manager is responsive to my requests for help or guidance”; “Top management in my business has a plan to improve the quality of our work and service.”

#### Turnover Intention

The authors referenced Mobley’s model of employees’ turnover process (Mobley, 1977) and Mitchel’s (1981) four-item scale to measure turnover intention. Sample items include “I am thinking of quitting”; “I would leave if I could find a better paying job.”

Empirical research shows that some demographic variables may be related to role stress, burnout and turnover. To avoid the possibility of spurious relationships and statistical confounds, gender, age, education, monthly income, occupation and tenure were elected as control variables in this study (e.g., Karatepe and Uludag, 2008; Kim and Lee, 2009; Jung et al., 2012).

### Procedure

Data for our study were collected through a field survey as well as internet questionnaire from 18 four-and-five-star hotels located in seven cities in South China. These hotels comprise international chains such as Marriott, Westin and Hilton and local chains such as Garden Hotel, Baiyun Guesthouse and Phoenix. The hotels range from resort hotels to exhibition and conference hotels. These hotels represent the types and sizes of high star hotels in southern China to some extent.

The participants were front-line employees in the hotel industry. We assured them that all information provided would be protected under strict confidentiality rules. We also told them there were no right or wrong answers. We collected data during two distinct periods. The first wave of data collection began on March 29 and ended on April 3, 2016, and the second wave took place from November 30 to December 11, 2017.

Seven of the 18 hotels agreed to participate in the field survey. With the cooperation of hotel managers, the respondents were chosen at ordered intervals according to employee identification numbers. In addition, five hotel administrations did not allow the authors to collect data from front-line employees directly. They arranged for a senior staff or supervisor to distribute the questionnaires among front-line employees. The authors went to the hotels several times. A total of 570 questionnaires were distributed, and 454 valid questionnaires were collected and analyzed for further analysis with an effective response rate of 79.6 percent. Eleven hotels participated in the internet questionnaire survey. With the assistance of supervisors of hotel departments, the authors received 129 usable responses.

The data were divided into two parts to conduct the pilot and main study. The pilot study, using 179 valid questionnaires, was conducted to clarify the dimensions of variables and confirm the reliability and validity of scales. The main study used 404 questionnaires to do confirmatory factor analysis and structural equation model analysis.

## Results

### Pilot Study

In the pilot study, the reliability of scales was measured by SPSS 21.0 through Cronbach’s coefficient alpha. The authors performed exploratory factor analysis to test the construct validity of the variables. In particular, all the estimated indices were above the threshold of 0.9 for Cronbach’s alpha (α role stress = 0.90, α burnout = 0.92, α service climate = 0.92, α turnover intention = 0.91), showing good consistency of the scales.

Through exploratory factor analysis, the authors extracted four fixed factors of role stress. The KMO was 0.87, and the four factors totally accounted for 64.61 percent of the variance after deleting three items (RC2, RO2, and RO5), indicating suitability for factor analysis. Hence, the authors inferred four dimensions of role stress: role conflict, role ambiguity, quantitative role overload and qualitative role overload. The KMO of burnout was 0.89, and the two factors explained the cumulative variability as 79.20 percent. The authors obtained two dimensions of burnout: emotional exhaustion and depersonalization.

### Main Study

In this study, we employed published scales, designed anonymous questionnaires, and arranged items pertaining to key constructs in separate parts (Conway and Lance, 2010). The authors varied the order of items, and designed three different versions of questionnaires to control the position bias to reduce CMV. Moreover, Harman’s one-factor test was performed to detect common method variance. The result shows that the first factor accounted for 28.74 percent of the variance, thus no serious CMV bias exists in this study, and confirmatory factor analyses also shows that the variables are distinct.

Once the authors verified the reliability of scales, the results showed that all the estimated indices were above the threshold of 0.70 for Cronbach’s alpha (range = 0.84 – 0.94), which indicates a rather high reliability of measurement scales. Confirmatory factor analysis was performed to assess the measurement model. The measurement model showed a good fit (χ*^2^* = 124.85, df = 46, χ*^2^*/df = 2.71, CFI = 0.98, IFI = 0.98, RMSEA = 0.05, SRMR = 0.06). An alternative one-factor model which combined all the items was specified, and showed a poorer fit (χ*^2^*/df = 9.27, CFI = 0.89, IFI = 0.89, RMSEA = 0.14, SRMR = 0.13). What’s more, the CRs of the various factors and variables were above 0.70 (range = 0.78 – 0.94). Except for the AVE of role conflict, the AVE values of the other factors and variables were all above 0.50 (range = 0.52 – 0.82), indicating that the scales has acceptable convergent validity (Table 1).

**TABLE 1:** Results of the confirmatory factor analysis.

Factors and		Standardized			
Variables	Items	Loading	CR	AVE	Cronbach’s α
Role Conflict			0.87	0.43	0.86
	RC1	0.48***			
	RC3	0.59***			
	RC4	0.46***			
	RC5	0.69***			
	RC6	0.71***			
	RC7	0.73***			
	RC8	0.68***			
	RC9	0.71***			
	RC10	0.75***			
Role Ambiguity			0.92	0.69	0.91
	RA1	0.75***			
	RA2	0.87***			
	RA3	0.87***			
	RA4	0.90***			
	RA5	0.77***			
Quantitative Role overload			0.78	0.55	0.79
	RO1	0.65***			
	RO3	0.73***			
	RO4	0.83***			
Qualitative Role overload			0.84	0.52	0.84
	RO6	0.64***			
	RO7	0.76***			
	RO8	0.86***			
	RO9	0.59***			
	RO10	0.73***			
Role Stress^a^			0.82	0.56	0.90
	Role Conflict	0.84***			
	Role Ambiguity	0.29**			
	Quantitative Role overload	0.77***			
	Qualitative Role overload	0.92***			
Emotional Exhaustion			0.93	0.73	0.94
	EE1	0.84***			
	EE2	0.83***			
	EE3	0.90***			
	EE4	0.85***			
	EE5	0.84***			
Depersonalization			0.94	0.79	0.93
	DE1	0.94***			
	DE2	0.93***			
	DE3	0.89***			
	DE4	0.77***			
Burnout^b^			0.90	0.82	0.94
	Emotional Exhaustion	0.92***			
	Depersonalization	0.89***			
Service Climate			0.93	0.65	0.94
	SC1	0.71***			
	SC2	0.76***			
	SC3	0.87***			
	SC4	0.91***			
	SC5	0.87***			
	SC6	0.89***			
	SC7	0.69***			
	SC8	0.76***			
Turnover Intention			0.92	0.75	0.93
	TI1	0.93***			
	TI2	0.93***			
	TI3	0.78***			
	TI4	0.81***			

**p < 0.01; ***p < 0.001. *χ*^2^ = 124.85, df = 46, *χ*^2^/df = 2.71, CFI = 0.98, IFI = 0.98, RMSEA = 0.05, SRMR = 0.06; Role Stress^*a*^ and Burnout^*b*^ indicators are derived from second-order factors.

Table 2 describes the mean values, standard deviations and Pearson correlations of key variables of this study. The results indicate significant correlations between dependent, mediating and independent variables. There is a significant positive correlation between role stress and burnout, confirming that the two dimensions of burnout are correlated positively with turnover intention.

**TABLE 2:** Correlation, mean, standard deviation.

Variables	Means	SD	RC	RA	ROa	ROb	EE	DE	SC	TI	RS	Burnout
RC	3.73	1.27	1.00									
RA	3.60	1.64	0.16**	1.00								
ROa	4.34	1.54	0.54**	0.17**	1.00							
ROb	3.58	1.33	0.67**	0.29**	0.55**	1.00						
EE	3.76	1.68	0.58**	0.20**	0.52**	0.64**	1.00					
DE	3.53	1.77	0.59**	0.15**	0.37**	0.59**	0.76**	1.00				
SC	4.61	1.34	−0.28**	−0.13**	−0.17**	−0.21**	−0.25**	−0.37**	1.00			
TI	4.29	1.81	0.43**	0.14**	0.28**	0.44**	0.49**	0.54**	−0.26**	1.00		
RS	3.81	1.06	0.77**	0.59**	0.77**	0.83**	0.64**	0.55**	−0.21**	0.43**	1.00	
Burnout	3.65	1.61	0.62**	0.18**	0.47**	0.66**	0.94**	0.94**	−0.33**	0.55**	0.64**	1.00

**p < 0.01. SD, standard deviation; RC, role conflict; RA, role ambiguity; ROa, quantitative role overload; ROb, qualitative role overload; EE, emotional exhaustion; DE, depersonalization; SC, service climate; TI, turnover intention; RS, role stress.

### The Mediating Effect of Burnout

In order to reduce the number of estimated parameters in structural equation modeling analysis as well as the influence of random errors and enhance stability of parameter estimation results, the authors adopted the parceling approach generally used by scholars when analyzing structural equation modeling (Bagozzi and Heatherton, 1994; Yang et al., 2010).

The authors built structural equation modeling to test the mediating effect of burnout on the relationship between role stress and employee turnover intention. The model appears to be acceptable (Table 3). A positive and significant relationship exists between role stress and burnout (β = 0.83, *p* < 0.001), providing evidence in favor of H1. Burnout has a significant positive effect on turnover intention (β = 0.62, *p* < 0.001), thus supporting H2. The mediating effect of burnout in the relationship between role stress and turnover intention was then investigated. The standardized path coefficient is 0.52, with a 95% confidence interval of [0.45, 0.59], indicating a significant indirect effect at *p* < 0.05 level. Thus, the results indicated that burnout has a significant mediating effect between role stress and turnover intention, supporting H3.

**TABLE 3:** Structural equation model results

Relationship	β	*T*-value
Role stress → Role conflict	0.75	16.24***
Role stress → Role ambiguity	0.28	6.24**
Role stress → Quantitative role overload	0.65	14.26***
Role stress → Qualitative role overload	0.76	–
Role stress → Burnout	0.83	15.52***
Burnout → Emotional exhaustion	0.85	–
Burnout → Depersonalization	0.82	20.39***
Burnout → Turnover Intention	0.62	13.99***

**p < 0.01; ***p < 0.001. *χ*^2^/df = 2.51, CFI = 0.98, IFI = 0.98, RMSEA = 0.06, SRMR = 0.08.

This study constructed a partial mediating model of the relationship between role stress and turnover intention to clarify whether the mediating effect of burnout is complete or partial. The results show that role stress is not significantly and positively related to turnover intention (β = 0.03, *p* = 0.64). At the same time, when the difference in degree of freedom is 1, the difference of χ*^2^* less than 3.84 (Δχ*^2^* = 0.09), indicating that the complete mediating model was better. Thus, burnout mediates the relationship between role stress and turnover intention fully.

### The Moderating Effect of Service Climate

As shown in Table 4, we regressed gender, age, education, income, occupation, tenure in the hotel industry, tenure in the current hotel and tenure in the current department. The results indicate that age has significant effects on turnover intention, income has negative effects on burnout, and occupation has significant effects on burnout and turnover intention. However, gender, education level, tenure in the hotel industry, in the current hotel and department are not significantly related to burnout and turnover intention.

**TABLE 4:** Results of moderated mediation.

Variables	Burnout				Turnover intention			
	M1	M2	M3	M4	M5	M6	M7	M8
Gender	0.06	–0.03	–0.03	–0.02	0.07	0.07	0.02	0.03
Age	–0.02	–0.09	–0.05	–0.05	–0.10	−0.11*	−0.15**	−0.11*
Education	0.03	–0.03	–0.04	–0.03	0.10	0.08	0.03	0.05
Monthly income	0.04	−0.08*	−0.07*	−0.06*	–0.04	–0.02	–0.09	–0.06
Occupation	0.20***	0.08**	0.04	0.05	0.25***	0.20***	0.11*	0.09
Tenure in hotel industry	–0.07	0.06	0.03	0.03	–0.05	–0.02	0.08	0.05
Tenure in current hotel	0.04	0.02	0.01	0.01	0.03	0.07	0.07	0.04
Tenure in current department	0.08	0.04	0.03	0.02	0.06	0.11	0.09	0.06
Role stress		0.62***	0.59***	0.60***			0.41***	0.13
Burnout								0.44***
Service climate			−0.19***	−0.19***				
Role stress* service climate				−0.11**				
Adj.R-square	0.04***	0.41***	0.44***	0.45**	0.06***	0.07*	0.22***	0.37***
Change R-square	0.04***	0.37***	0.03***	0.01**	0.06***	0.01*	0.15***	0.15***
*F*	16.52***	25.21***	24.58***	7.94**	26.62***	15.56***	39.60***	51.98***

*p < 0.05; **p < 0.01; ***p < 0.001.

Then, we regressed the main effects of role stress and service climate to burnout in models 2 and 3 of the analysis, respectively. The results show that the coefficient of independent variable role stress is significant (β = 0.62, *p* < 0.001, *R*^2^ = 0.41, Model 2), and the coefficient of moderating variable service climate is significant (β = −0.19, *p* < 0.001, *R*^2^ = 0.44, Model 3). When studying the effect of service climate, we entered the interaction between role stress and service climate in Model 4. The interaction term is negatively related to burnout (β = −0.11, *p* < 0.01, *R*^2^ = 0.45, Model 4), indicating that the moderating effect of the service climate is significant, supporting H4 (Table 4).

**FIGURE 2:**
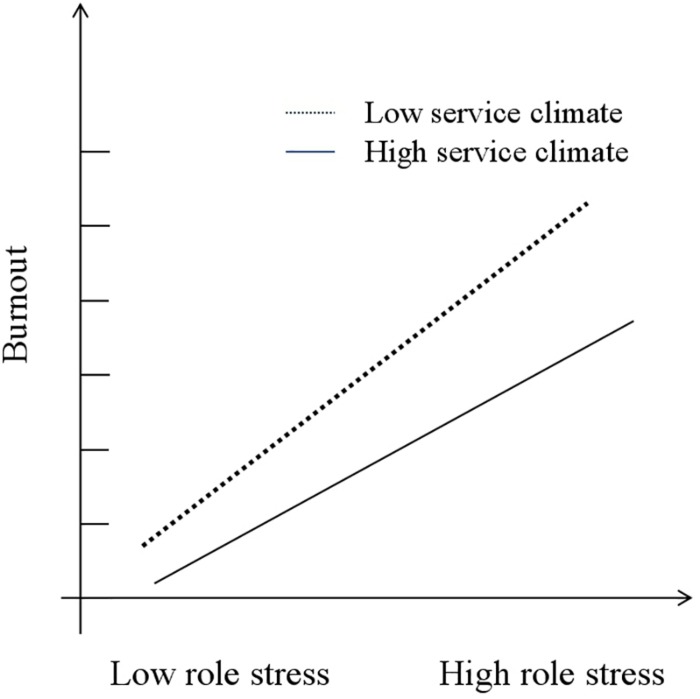
Moderating effect of service climate.

After taking the average value as a standard, the 404 samples were divided into two groups depending on employee perception of service climate. The perceived low-service-climate group has 189 samples, while the perceived high-service-climate group has 215 samples. The authors conducted a moderation analysis based on the two groups. As shown in Figure 2, in the perceived low-service-climate group, burnout was higher, and with increased role stress, burnout tends to increase more rapidly. Compared with the lower group, in another group, burnout was lower, and with the increase of role stress, employees’ burnout increased more slowly.

Moderated path analysis (Edwards and Lambert, 2007) was performed to test H5 (moderated-mediation). As shown in Table 5, the indirect effect of role stress on turnover intention via burnout varied significantly across different levels of service climate. Specifically, the indirect effect of RS on TI via JB was stronger when the service climate was low (β = 0.54, *p* < 0.001) rather than high (β = 0.41, *p* < 0.001). Hence, H5 was supported.

**TABLE 5:** Results of the moderated path analysis.

Moderator variable	RS (X) → JB (M) → TI (Y)				
	Stage		Effect		
	First	Second	Direct	Indirect	Total
	P_MX_	P_YM_	(P_YX_)	(P_YM_ P_MX_)	(P_YX_ + P_YM_ P_MX_)
High service climate	0.80***	0.51***	0.19*	0.41***	0.60***
Low service climate	1.05***	0.51***	0.19*	0.54***	0.73***
Differences	−0.25***	0.00	−0.01*	−0.13**	−0.13**

*p < 0.05; **p < 0.01; ***p < 0.001. P_*MX*_, path from RS to JB; P_*YM*_, path from JB to TI; P_*YX*_, path from RS to TI; Tests of differences for the indirect and total effect were based on bias-corrected confidence intervals derived from bootstrap estimates.

According to the literature, the authors classify role conflict and role ambiguity as hindrances and quantitative as well as qualitative role overload as challenges. To further understand the moderating effect on challenge-hindrance stressors, we build structural equation modeling to test the relationship among variables and test the moderating effect of service climate (Table 6).

**TABLE 6:** Results of structural equation modeling.

Relationship	β	*T*-value
Hindrance stressors → Role conflict	0.70	12.31***
Hindrance stressors → Role ambiguity	0.27	–
Challenge stressors → Quantitative role overload	0.77	14.26***
Challenge stressors → Qualitative role overload	0.91	–
Hindrance stressors → Burnout	0.39	4.11**
Challenge stressors → Burnout	0.62	10.47***
Burnout → Turnover intention	0.58	9.59***

**p < 0.01; ***p < 0.001. χ*^2^*/df = 2.86, CFI = 0.98, IFI = 0.98, RMSEA = 0.07, SRMR = 0.07.

The model appears to be acceptable. Positive and significant relationships exist between hindrance stressors and burnout (β = 0.39, *p* < 0.01) as well as challenge stressors and burnout (β = 0.62, *p* < 0.001). Burnout has a significant positive effect on turnover intention (β = 0.58, *p* < 0.001). In addition, burnout mediates the relationship between hindrance and challenge stressors and turnover intention significantly. Moreover, according to the moderated path analysis results, the interaction between hindrance stressors and service climate was negatively related to burnout (β = −0.10, *p* < 0.01) while the interaction between challenge stressors and service climate was not significantly related to burnout (β = −0.01, *p* = 0.87).

## Discussion and Conclusion

### Discussion and Theoretical Implications

This study adds to the stress and service management literature in several ways. First, the study’s findings contribute to existing knowledge about role stress by providing empirical support to understand hospitality employees’ role stress. Previous research has examined the impact of role stress on employees from a variety of industry settings, including salespersons, nurses, engineers and administration staff (Schaubroeck et al., 1989; Bacharach et al., 1991; Jones et al., 2007). Front-line employees in hospitality industry are also under serious role stress, which exerts detrimental impacts on employees and hospitality organizations. However, research isolating the dimensions of role overload and further investigating the effects of role overload on employees’ psychological and behavioral outcomes in hospitality settings is scarce.

In previous studies, the view that role stress is a two-dimensional concept does not consider role overload as an essential dimension. Some scholars suggest that role stress contains three dimensions, arguing role overload is the third independent dimension. This study contributes to this stream of research not only by confirming role overload exists as an essential dimension of role stress, but also by further identifying the sub-dimensions of role overload. The results of confirmatory factor analysis in this study demonstrate that qualitative role overload and quantitative role overload are two separate aspects which can be seen as two stable dimensions. By comparing the square root of AVE of each dimension of role stress to the correlations of these dimensions, the authors find that the square root of AVE of ROa is higher than the correlations among ROa, Rob and other dimensions of role stress. Moreover, the model fits better of four-dimensional structure than three-dimensional. Thus, this study suggests that overload is differentiable from role conflict and ambiguity, and investigation affirms the validity of role overload as a construct distinct from role conflict. The authors consider that qualitative role overload and quantitative role overload as two substantive parts, and argue that role stress has four stable dimensions: role conflict, role ambiguity, qualitative role overload and quantitative role overload. Consequently, it is feasible to use a four-dimensional structure in the hotel industry when exploring front-line employee role stress.

Second, this study advances understanding of how role stress lead to employee turnover intention. Accumulated employees stress may cause burnout, which manifests as various physical, behavioral, and psychological symptoms. Through structural equation modeling, the authors find that burnout fully mediates the relationship between role stress and turnover intention completely. In other words, rRole stress indirectly affects turnover intent through burnout, revealing that stressed employees do not choose to resign immediately unless they suffer from a high level of burnout. The results findings echo with previous literature (e.g., Ahuja et al., 2007; Kim and Stoner, 2008; Mulki et al., 2008; Kim and Lee’s, 2009), which proves that stress does not directly lead to turnover, and an appropriate and tolerable level of stress may not generate negative effects (Beehr and Newman, 1978). In line with previous literature, this study indicates that burnout is the main and direct reason for employee turnover (Singh et al., 1994; Fogarty et al., 2000; Schaufeli and Bakker, 2004).

Third, prior studies of service climate have primarily focused on settings other than hotels (e.g., Teng and Barrows, 2009; Jung and Yoon, 2013). This study points to the potential importance of research on service climate in hotel settings. Service climate is an important linkage when converting organizational resources and management concept to practical performance (Lin and Liu, 2016). In this research, service climate was brought into the relationship as a moderator that can be viewed as an enrichment to the role stress, burnout and employee turnover models. The authors explore its moderating effect on the relationship between role stress and front-line employee burnout in the hotel industry. Service climate is found to be a critical variable to coordinate the relation between role stress and burnout. Literature suggests that good service climate can positively influence employee perceptions and work attitudes, and have a moderating effect on stress consequences (Arasli et al., 2017). Beyond what is well-researched in literature, this study reveals that a good service climate could significantly reduce the negative effect of role stress on burnout. The findings of this study further assure the role of service climate in the hospitality industry, showing the necessity of a broad discussion about the interrelations between service climate and employee behaviors.

Finally, this study offers some enlightening results about challenge-hindrance stressors. Research on hindrance and challenge stressors is expanding, although debate continues on the effects of these two types of stressors on job-related outcomes. Our empirical results show that both hindrance and challenge stressors lead to burnout. An unexpected finding of our research is that the interaction between hindrance stressors and service climate has a negative effect on burnout, while the interaction between challenge stressors and service climate is not significantly related to burnout. The findings reveal the boundary condition of service climate’s moderating effect on burnout. Specifically, the results indicate that improving service climate as an intervention to alleviate the negative effects of role stress is only effective when the stressors are hindrance. This specific finding has two implications. For one thing, by identifying different effects of challenge and hindrance stressors, this study provides supporting evidence to the distinctive nature of these two stress types (Lepine et al., 2005; Webster et al., 2011). For another, in previous literature, researchers agree that challenge stressor may not necessarily be bad while hindrance stressor is often detrimental (Cavanaugh et al., 2000). This study adds more knowledge on understanding hindrance stressor, demonstrating that there actually is a solution to mitigate the negative effect of hindrance stressor by creating a positive service climate.

### Managerial Implications

Our research has the following implications for executives and managers of hotels. First, conflicts and work overload are endemic among hotel front-line employees. Most of the front-line employees are in a stable working group. They identify with the rationality of their job and have clear ideas about their responsibilities. The main reason behind role conflict is that people’s requirements and expectations vary with who they are as individuals. Superiors should define kinds of duties of employees when they allocate assignments, and try to eliminate extra work as much as possible to reduce the feelings of role conflict.

The authors find that most staff think their job is relatively simple and within their capacity. However, the workload is so broad that they have to sacrifice their rest time to catch up with the work. In this situation, it is necessary for all departments to clarify the allocation of tasks and plan the operational and organizational structures as well as the shift system in order to improve productivity and minimize unnecessary extra working hours. Hotel managers should regularly check frontline employees’ stress level. If their stress reaches a threshold situation, managers could allow them to leave and recharge.

According to several surveys (Singh et al., 1994; Schaufeli and Bakker, 2004), it is normal for employees to feel jaded, which leads to resignation and has a negative effect on service quality. Front-line employees do their routine jobs, receive poor pay, and often feel a sense of emotional exhaustion. Therefore, managers need to address the range of emotions in their staffs. A variety of extracurricular activities, training and competition can be organized to avoid negative impacts on both the employees and enterprises. Besides, many hotel employees are uncertain about the value of their jobs, which also happens in other industries and is related to changing social values. Most front-line employees are young, with more than half of them under the age of 28, and they have not yet developed a clear career plan. Thus, more career planning should be provided to help front-line employees gain thorough insights into the hotel business and find their own career paths.

Second, pressure is a double-edged sword. A certain amount of pressure may stimulate the enthusiasm of employees, push them to complete tasks and realize their career goals (Karatepe and Uludag, 2008). Conversely, too much pressure may discourage them from trying their utmost in their work. It is not role stress but burnout that directly leads to turnover (Fogarty et al., 2000; Kim and Stoner, 2008; Kim and Lee, 2009). This study suggests hotel managers proceed to reduce turnover intention in two directions. Hotels need to reduce employees’ perception of pressure to decrease burnout. Training programs on how to monitor and regulate emotional exhaustion as well as depersonalization could be offered to front-line employees. As a result of training programs, employees may learn more about self-regulation strategies. It is important to note that measures to control the buffer factor that minimizes the influence of role stress or burnout are essential. Participation in management, social support and other factors can regulate the relationship between role stress and its dependent variables.

Third, service climate is another variable that affects role stress, burnout and turnover intention. The authors suggest that hotels can improve the organizational service climate through communication, incentive schemes, service quality and corporate responsibility plans. Hotels can provide training to help front-line employees expand their knowledge. At the same time, hotels should reflect professional attitudes and high standards with regard to training employees. They can also provide training in service improvement and organization optimization in addition to offering incentives that emphasize the relationship between employees’ psychological status and the development of hotels. As the service climate improves. employees may feel more attached to their hotels, which may lead to further improvements in the service climate (Arasli et al., 2017). Hotels need to establish fair, open and effective incentive schemes so that each employee knows he can expect rewards for good work. Hotels need to offer employee assistance programs, which may contribute to relieving stress and helping employees to reach high service performance. Moreover, hotels are advised to encourage communication between employees and their superiors and colleagues to express stressful and negative emotions, and share work skills and suggestions, provide psychological consultations to eliminate stress.

Enhancing service quality is a systematic project. The administration should lead by example through providing quality service to customers and staff and helping employees achieve higher job satisfaction. In this research sample, more than half of front-line staffers are college students who go to work for hotels as soon as they graduate. Hotels can give their employees opportunities to participate in social activities while promoting healthy lifestyles to help them develop a positive attitude to life. When employees have turnover intentions, hotels should not only seek reasons from their employees, but reflect whether they provide a good service to their employees.

### Limitations and Future Research

There are some limitations that may be considered as opportunities for future research. The paper examined 18 hotels in South China. Considering cultural differences, future research efforts could be exerted on generalizing the findings of this study or expanding the research model in different cultural contexts. Next, this study demonstrates that qualitative and quantitative role overload are two stable dimensions of role stress in hospitality settings. Future studies could use the four-dimensional scale to measure front-line employee role stress, and further validate the scale. In addition, this article confirms that service climate is a moderating variable to mitigate the negative effects of role stress on burnout. The authors suggest that other organizational factors be included in the model, such as leader-member exchange and organizational justice. Future studies could take these variables into account to further explore the impacts of organizational factors. Moreover, not all stress is bad, and the boundary between “good” and “bad” role stress is worth discussing in the future. Since the results of this study indicate that service climate has different influences on hindrance and challenge stressors, future studies could explore other appropriate moderators for challenge stressors. Finally, the authors would also suggest adopting longitudinal data for examining dynamic variables like stress and burnout in future studies since these variables change over time.

## Data Availability Statement

The datasets generated for this study are available on request to the corresponding author.

## Author Contributions

BW and XMZ conceptualized the research. BW, XMZ, and XZ collected the data, which were analyzed by XMZ. XMZ and YH prepared the manuscript. BW and YH provided the critical feedback.

## Conflict of Interest

The authors declare that the research was conducted in the absence of any commercial or financial relationships that could be construed as a potential conflict of interest.

## References

[B1] Abdel-Halim A. A. (1981). Effects of role stress-job design-technology interaction on employee work satisfaction. *Acad. Manag. J.* 24 260–273. 10.2307/255840

[B2] Ahuja M. K. Chudoba K. M. Kacmar C. J. Mcknight D. H. George J. F. (2007). IT road warriors: balancing work-family conflict, job autonomy, and work overload to mitigate turnover intentions. *MIS Q.* 31 1–17. 10.2307/25148778

[B3] Arasli H. Teimouri R. B. Kiliç H. Aghaei I. (2017). Effects of service orientation on job embeddedness in hotel industry. *Serv. Ind. J.* 37 607–627. 10.1080/02642069.2017.1349756

[B4] Ayşe Banu E. Alexander Ellinger E. (2018). Alleviating job stress to improve service employee work affect: the influence of rewarding. *Serv. Bus.* 12 121–141. 10.1007/s11628-017-0340-y

[B5] Bacharach S. B. Bamberger P. Conley S. (1991). Work-home conflict among nurses and engineers: mediating the impact of role stress on burnout and satisfaction at work. *J. Organ. Behav.* 12 39–53. 10.1002/job.4030120104

[B6] Bagozzi R. P. Heatherton T. F. (1994). A general approach to representing multifaceted personality constructs: application to state self-esteem. *Struct. Equ. Modeling* 1 35–67. 10.1080/10705519409539961

[B7] Beehr T. A. Newman J. E. (1978). Job stress, employee health, and organizational effectiveness: a facet analysis, model, and literature review. *Pers. Psychol.* 31 665–669. 10.1111/j.1744-6570.1978.tb02118.x

[B8] Bliese P. D. Jex S. M. (2002). Incorporating a mulitilevel perspective into occupational stress research: theoretical, methodological, and practical implications. *J. Occup. Health Psychol.* 7 265–276. 10.1037/1076-8998.7.3.265 12148957

[B9] Brookings J. B. Bolton B. Brown C. E. Mcevoy A. (1985). Self-reported job burnout among female human service professionals. *J. Organ. Behav.* 6 143–150. 10.1002/job.4030060205 25619652

[B10] Büssing A. Glaser J. (2000). Four-stage process model of the core factors of burnout: the role of work stressors and work-related resources. *Work Stress* 14 329–346. 10.1080/02678370110041884

[B11] Cable D. M. Parsons C. K. (2001). Socialization tactics and person-organization fit. *Pers. Psychol.* 54 1–23. 10.1111/j.1744-6570.2001.tb00083.x 15769234

[B12] Cavanaugh M. A. Boswell W. R. Roehling M. V. Boudreau J. W. (2000). An empirical examination of self-reported work stress among U.S. managers. *J. Appl. Psychol.* 85 65–74. 10.1037/0021-9010.85.1.65 10740957

[B13] Chang K. C. (2016). Effect of services cape on customer behavioral intentions: moderating roles of service climate and employee engagement. *Int. J. Hosp. Manag.* 53 116–128. 10.1016/j.ijhm.2015.12.003

[B14] Chen J. Wang L. Tang N. (2016). Half the sky: the moderating role of cultural collectivism in job turnover among Chinese female workers. *J. Bus. Ethics* 133 1–12. 10.1007/s10551-014-2395-1

[B15] Cho S. Johanson M. M. Guchait P. (2009). Employees intent to leave: a comparison of determinants of intent to leave versus intent to stay. *Int. J. Hosp. Manag.* 28 374–381. 10.1016/j.ijhm.2008.10.007

[B16] Choi H. M. Mohammad A. A. A. Kim W. G. (2019). Understanding hotel frontline employees’ emotional intelligence, emotional labor, job stress, coping strategies and burnout. *Int. J. Hosp. Manag.* 82 199–208. 10.1016/j.ijhm.2019.05.002

[B17] Conway J. M. Lance C. E. (2010). What reviewers should expect from authors regarding common method bias in organizational research. *J. Bus. Psychol.* 25 325–334. 10.1007/s10869-010-9181-6

[B18] Cooper C. L. Marshall J. (1976). Occupational sources of stress: a review of the literature relating to coronary heart disease and mental ill-health. *J. Occup. Psychol.* 49 11–28. 10.1111/j.2044-8325.1976.tb00325.x

[B19] Coverman S. (1989). Role overload, role conflict and stress: addressing consequences of multiple role demands. *Soc. Forces* 67 965–982. 10.2307/2579710

[B20] Crawford E. R. Lepine J. A. Rich B. L. (2010). Linking job demands and resources to employee engagement and burnout: a theoretical extension and meta-analytic test. *J. Appl. Psychol.* 95 834–848. 10.1037/a0019364 20836586

[B21] Darvishmotevali M. Arasli H. Kilic H. (2017). Effect of job insecurity on frontline employee’s performance: looking through the lens of psychological strains and leverages. *Int. J. Contemp. Hosp. Manag.* 29 1724–1744. 10.1108/IJCHM-12-2015-0683

[B22] Demerouti E. Nachreiner F. Bakker A. B. Schaufeli W. B. (2001). The job demands-resources model of burnout. *J. Appl. Psychol.* 86 499–512. 10.1037/0021-9010.86.3.499 11419809

[B23] Densten I. L. (2001). Re-thinking burnout. *J. Organ. Behav.* 22 833–847. 10.1002/job.115

[B24] Edwards J. R. Lambert L. S. (2007). Methods for integrating moderation and mediation: a general analytical framework using moderated path analysis. *Psychol. Methods* 12 1–22. 10.1037/1082-989X.12.1.1 17402809

[B25] Fogarty T. J. Singh J. Rhoads G. K. Moore R. K. (2000). Antecedents and consequences of burnout in accounting: beyond the role stress model. *Behav. Res. Account.* 12 31–67. 10.1504/EJIM.2019.10017013

[B26] Freudenberger H. J. (1974). Staff burn-out. *J. Soc. Issues* 30 159–165. 10.1111/j.1540-4560.1974.tb00706.x

[B27] Hecht L. M. (2001). Role conflict and role overload: different concepts, different consequences. *Sociol. Inq.* 71 111–121. 10.1111/j.1475-682X.2001.tb00930.x

[B28] Hong Y. Liao H. Hu J. Jiang K. (2013). Missing link in the service profit chain: a meta-analytic review of the antecedents, consequences, and moderators of service climate. *J. Appl. Psychol.* 98 237–267. 10.1037/a0031666 23458337

[B29] Huang S. S. Veen R. V. D. Song Z. C. (2018). The impact of coping strategies on occupational stress and turnover intentions among hotel employee. *J. Hosp. Mark. Manag.* 27 926–945. 10.1080/19368623.2018.1471434

[B30] Ivancevich J. M. Matteson M. T. (1980). *Stress and Work: A Managerial Perspective.* Glenview, IL: Scott Foresman.

[B31] Iverson R. D. Olekalns M. Erwin P. J. (1998). Affectivity, organizational stressors, and absenteeism: a causal model of burnout and its consequences. *J. Vocat. Behav.* 52 1–23. 10.1006/jvbe.1996.1556

[B32] Jaramillo F. Mulki J. P. Boles J. S. (2011). Workplace stressors, job attitude, and job behaviors: is interpersonal conflict the missing link? *J. Pers. Sell. Sales Manag.* 31 339–356. 10.2753/pss0885-3134310310

[B33] Jaramillo F. Mulki J. P. Solomon P. (2006). The role of ethical climate on salesperson’s role stress, job attitudes, turnover intention, and job performance. *J. Pers. Sell. Sales Manag.* 26 271–282. 10.2753/PSS0885-3134310310

[B34] Johnson J. W. (1996). Linking employee perceptions of service climate to customer satisfaction. *Pers. Psychol.* 49 831–851. 10.1111/j.1744-6570.1996.tb02451.x 12002951

[B35] Jones E. Chonko L. Rangarajan D. Roberts J. (2007). The role of overload on job attitudes, turnover intentions, and salesperson performance. *J. Bus. Res.* 60 663–671. 10.1016/j.jbusres.2007.02.014

[B36] Jung H. S. Yoon H. H. (2013). The effects of organizational service orientation on person-organization fit and turnover intent in a deluxe hotel. *Serv. Ind. J.* 33 7–29. 10.1080/02642069.2011.596932

[B37] Jung H. S. Yoon H. H. Kim Y. J. (2012). Effects of culinary employees’ role stress on burnout and turnover intention in hotel industry: moderating effects on employees’ tenure. *Serv. Ind. J.* 32 2145–2165. 10.1080/02642069.2011.574277

[B38] Kahn R. L. Wolfe D. M. Quinn R. P. Snoek J. D. Rosenthal R. A. (1964). *Occupational Stress: Studies in Role Conflict and Ambiguity.* New York, NY: John Wiley & Sons.

[B39] Karatepe O. M. (2013). The effects of work overload and work-family conflict on job embeddedness and job performance: the mediation of emotional exhaustion. *Int. J. Contemp. Hosp. Manag.* 25 614–634. 10.1108/09596111311322952

[B40] Karatepe O. M. Beirami E. Bouzari M. Safavi H. P. (2014). Does work engagement mediate the effects of challenge stressors on job outcomes? Evidence from the hotel industry. *Int. J. Hosp. Manag.* 36 14–22. 10.1016/j.ijhm.2013.08.003

[B41] Karatepe O. M. Karatepe T. (2010). Role stress, emotional exhaustion, and turnover intentions: does organizational tenure in hotels matter? *J. Hum. Resour. Hosp. Tour.* 9 1–16. 10.1080/15332840903323364

[B42] Karatepe O. M. Uludag O. (2008). Role stress, burnout and their effects on frontline hotel employees’ job performance: evidence from Northern Cyprus. *Int. J. Tour. Res.* 10 111–126. 10.1002/jtr.645

[B43] Karatepe O. M. Yavas U. Babakus E. Deitzc G. D. (2018). The effects of organizational and personal resources on stress, engagement, and job outcomes. *Int. J. Hosp. Manag.* 74 147–161. 10.1016/j.ijhm.2018.04.005

[B44] Kelly T. Barrett M. (2011). The leading causes and potential consequences of occupational stress: a study of Irish trainee accountants. *Ir. Account. Rev.* 18 31–55.

[B45] Kemery E. R. Bedeian A. G. Mossholder K. W. Touliatos J. (1985). Outcomes of role stress: a multisample constructive replication. *Acad. Manag. J.* 28 363–375. 10.5465/256206 10272105

[B46] Kilroy S. Flood P. C. Bosak J. Chênevert D. (2016). Perceptions of high-involvement work practices and burnout: the mediating role of job demands. *Hum. Resour. Manag. J.* 26 408–424. 10.1111/1748-8583.12112

[B47] Kim H. Lee S. Y. (2009). Supervisory communication, burnout, and turnover intention among social workers in health care settings. *Soc. Work Health Care* 48 364–385. 10.1080/00981380802598499 19396707

[B48] Kim H. Stoner M. (2008). Burnout and turnover intention among social workers: effects of role stress, job autonomy and social support. *Adm. Soc. Work* 32 5–25. 10.1080/03643100801922357

[B49] Kim S. M. Um K. H. Kim H. Y. Kim Y. H. (2016). Hospital career management systems and their effects on the psychological state and career attitudes of nurses. *Serv. Bus.* 10 87–112. 10.1007/s11628-014-0257-7

[B50] Kim S. S. Im J. Hwang J. (2015). The effects of mentoring on role stress, job attitude, and turnover intention in the hotel industry. *Int. J. Hosp. Manag.* 48 68–82. 10.1016/j.ijhm.2015.04.006

[B51] Kim T. T. Paek S. Chang H. C. Lee G. (2012). Frontline service employees’ customer-related social stressors, emotional exhaustion, and service recovery performance: customer orientation as a moderator. *Serv. Bus.* 6 503–526. 10.1007/s11628-012-0164-8

[B52] Kim W. G. Choi H. M. Li J. (2016). Antecedents and outcomes of migrant workers’ sociocultural adjustment in the hospitality industry. *Int. J. Hosp. Manag.* 58 1–12. 10.1016/j.ijhm.2016.06.009

[B53] Konstantinou A. K. Bonotis K. Maria S. Vasileios S. Dardiotis E. (2018). Burnout evaluation and potential predictors in a Greek Cohort of mental health nurses. *Arch. Psychiatr. Nurs.* 32 449–456. 10.1016/j.apnu.2018.01.002 29784229

[B54] Kralj A. Solnet D. (2010). Service climate and customer satisfaction in a casino hotel: an exploratory case study. *Int. J. Hosp. Manag.* 29 711–719. 10.1016/j.ijhm.2010.01.005

[B55] Kristof A. L. (1996). Person-organization fit: an integrative review of its conceptualizations, measurement, and implications. *Pers. Psychol.* 49 1–49. 10.1111/j.1744-6570.1996.tb01790.x

[B56] Kuo H. T. Lin K. C. Li I. C. (2014). The mediating effects of job satisfaction on turnover intention for long-term care nurses in Taiwan. *J. Nurs. Manag.* 22 225–233. 10.1111/jonm.12044 23465339

[B57] Lazarus R. S. Folkman S. (1984). *Stress, Appraisal, and Coping.* New York, NY: Springer.

[B58] Lee R. T. Ashforth B. E. (1996). A meta-analytic examination of the correlates of the three dimensions of job burnout. *J. Appl. Psychol.* 81 123–133. 10.1037/0021-9010.81.2.123 8603909

[B59] Lepine J. A. Podsakoff N. P. Lepine M. A. (2005). A meta-analytic test of the challenge stressor-hindrance stressor framework: an explanation for inconsistent relationships among stressors and performance. *Acad. Manag. J.* 48 764–775. 10.5465/amj.2005.18803921

[B60] Lin Y. T. Liu N. C. (2016). High performance work systems and organizational service performance: the roles of different organizational climates. *Int. J. Hosp. Manag.* 55 118–128. 10.1016/j.ijhm.2016.04.005

[B61] Lingard H. (2010). The impact of individual and job characteristics on‘burnout’ among civil engineers in Australia and the implications for employee turnover. *Constr. Manag. Econ.* 21 69–80. 10.1080/0144619032000065126

[B62] Magnano P. Santisi G. Platania S. (2017). Emotional intelligence as mediator between burnout and organisational outcomes. *Int. J. Work Organ. Emot.* 8 305–320. 10.1504/IJWOE.2017.089295

[B63] Maslach C. Jackson S. E. (1984). Burnout in organizational settings. *Appl. Soc. Psychol. Annu.* 5 133–153. 10.4236/ojs.2015.57065

[B64] Maslach C. Schaufeli W. B. Leiter M. P. (2001). Job burnout. *Annu. Rev. Psychol.* 52 397–422. 10.1146/annurev.psych.52.1.397 11148311

[B65] Mérida-López S. Extremera N. (2017). Emotional intelligence and teacher burnout: a systematic review. *Int. J. Educ. Res.* 85 121–130. 10.1016/j.ijer.2017.07.006 25851427

[B66] Mitchel J. O. (1981). The effect of intentions, tenure, personal, and organizational variables on managerial turnover. *Acad. Manag. J.* 24 742–750. 10.5465/256173

[B67] Mobley W. H. (1977). Intermediate linkages in the relationship between job satisfaction and employee turnover. *J. Appl. Psychol.* 62 237–240. 10.1037/0021-9010.62.2.237

[B68] Mohsin A. Lengler J. Kumar B. (2013). Exploring the antecedents of intentions to leave the job: the case of luxury hotel staff. *Int. J. Hosp. Manag.* 35 48–58. 10.1016/j.ijhm.2013.05.002

[B69] Montani F. Courcy F. Vandenberghe C. (2017). Innovating under stress: the role of commitment and leader-member exchange. *J. Bus. Res.* 77 1–13. 10.1016/j.jbusres.2017.03.024

[B70] Mulki J. P. Jaramillo J. F. Locander W. B. (2008). Effect of ethical climate on turnover intention: linking attitudinal and stress theory. *J. Bus. Ethics* 78 559–574. 10.1007/s10551-007-9368-6

[B71] O’ Neill J. W. Davis K. (2011). Work stress and well-being in the hotel industry. *Int. J. Hosp. Manag.* 30 385–390. 10.1016/j.ijhm.2010.07.007 23794780 PMC3686125

[B72] Peterson M. F. Smith P. B. Akande A. Ayestaran S. Bochner S. Callan V. (1995). Role conflict, ambiguity, and overload: a 21-nation study. *Acad. Manag. J.* 38 429–452. 10.5465/256687

[B73] Rizzo J. R. House R. J. Lirtzman S. I. (1970). Role conflict and ambiguity in complex organizations. *Adm. Sci. Q.* 15 150–163. 10.2307/2391486

[B74] Schaubroeck J. Cotton J. L. Jennings K. R. (1989). Antecedents and consequences of role stress: a covariance structure analysis. *J. Organ. Behav.* 10 35–58. 10.1002/job.4030100104

[B75] Schaufeli W. B. Bakker A. B. (2004). Job demands, job resources, and their relationship with burnout and engagement: a multi-sample study. *J. Organ. Behav.* 25 293–315. 10.1002/job.248

[B76] Schaufeli W. B. Leiter M. P. Maslach C. Jackson S. E. (1996). “Maslach burnout inventory – general survey”, in *The Maslach Burnout Inventor—Test Manual*, 3rd Edn, eds Jackson C. Maslach S. E. Leiter M. P. (Palo Alto, CA: Consulting Psychologists Press).

[B77] Schneider B. White S. S. Paul M. C. (1998). Linking service climate and customer perceptions of service quality: test of a causal model. *J. Appl. Psychol.* 83 150–163. 10.1037/0021-9010.83.2.150 9577232

[B78] Singh J. (1993). Boundary role ambiguity: facets, determinants, and impacts. *J. Mark.* 57 11–31. 10.1177/002224299305700202

[B79] Singh J. Goolsby J. R. Rhoads G. K. (1994). Behavioral and psychological consequences of boundary spanning burnout for customer service representatives. *J. Mark. Res.* 31 558–569. 10.1177/002224379403100409

[B80] Teng C. C. Barrows C. W. (2009). Service orientation: antecedents, outcomes, and implications for hospitality research and practice. *Serv. Ind. J.* 29 1413–1435. 10.1080/02642060903026247

[B81] Teoh M. W. Wang Y. Kwek A. (2019). Coping with emotional labor in high stress hospitality work environments. *J. Hosp. Mark. Manag.* 28 883–904. 10.1080/19368623.2019.1571979

[B82] Thomas C. H. Lankau M. J. (2009). Preventing burnout: the effects of lmx and mentoring on socialization, role stress, and burnout. *Hum. Resour. Manag.* 48 417–432. 10.1002/hrm.20288

[B83] Walker O. C. Churchill G. A. Ford N. M. (1975). Organizational determinants of the industrial salesman’s role conflict and ambiguity. *J. Mark.* 39 32–39. 10.1177/002224297503900106

[B84] Webster J. R. Beehr T. A. Love K. (2011). Extending the challenge-hindrance model of occupational stress: the role of appraisal. *J. Vocat. Behav.* 79 505–516. 10.1016/j.jvb.2011.02.001

[B85] Wirtz J. Jerger C. (2016). Managing service employees: literature review, expert opinions, and research directions. *Serv. Ind. J.* 36 757–788. 10.1080/02642069.2016.1278432

[B86] Yang C. Nay S. Hoyle R. H. (2010). Three approaches to using lengthy ordinal scales in structural equation models: parceling, latent scoring, and shortening scales. *Appl. Psychol. Meas.* 34 122–142. 10.1177/0146621609338592 20514149 PMC2877522

